# Tyrosine Kinase Inhibitor Therapy Discontinuation for Patients with Chronic Myeloid Leukaemia in Clinical Practice

**DOI:** 10.1007/s11899-019-00548-2

**Published:** 2019-11-07

**Authors:** Richard E. Clark

**Affiliations:** Department of Molecular and Clinical Cancer Medicine, Institute of Translational Medicine, University of Liverpool, Room 150, First floor, Sherrington Building, Ashton Street, Liverpool,, L69 3GE UK

**Keywords:** Chronic myeloid leukaemia, CML, Discontinuing treatment, TKI, Tyrosine kinase inhibitors

## Abstract

**Purpose:**

In chronic myeloid leukaemia, tyrosine kinase inhibitor treatment is traditionally given continuously for life. However, these drugs produce excellent responses for many patients, and this is accompanied by survival that is close to normal. This has prompted studies of whether it is possible to stop treatment, thus achieving a treatment-free remission (TFR).

**Recent Findings:**

Most TFR studies have focussed on abrupt cessation in patients with long-standing deep remissions, but recent data suggest that more gradual treatment de-escalation may improve TFR success, and that it may be possible to extend TFR attempts to patients who are in stable major molecular response but not necessarily MR4.

**Summary:**

Further data are badly needed on TFR for patients whose remission is less than stable MR4 and on the importance of prior interferon-alpha treatment. Funding TFR trials in a disease with such an excellent outlook is an increasing challenge.

## Introduction

Myelosuppressive therapy in the form of busulphan was introduced for the treatment of chronic myeloid leukaemia (CML) in the early 1950s, and it and its more convenient successor hydroxycarbamide remained the centrepiece of CML therapy for the next 30 years. It was assumed that these agents should be given continually, perhaps in the belief that it was important to sustain suppression of an overactive marrow. Trials were done to examine the optimal timing of busulphan delivery (reviewed in [1]), but none considered whether it might be feasible to stop treatment in good responders, presumably because the Philadelphia (Ph) t(9:22)(q34:q11) translocation that typifies CML remains readily detectable in the marrow of even the best responders to busulphan/hydroxycarbamide. Interferon-alpha (IFN) became available in the early 1980s, and serial cytogenetic studies in IFN recipients revealed for the first time that around half could achieve some degree of marrow cytogenetic remission. A small minority (~ 5%) of patients may achieve enduring complete cytogenetic remission (CCyR; none of at least 20 marrow metaphases are Ph positive), and about half the patients in long-standing CCyR on IFN may successfully stop treatment [2].

The tyrosine kinase inhibitor (TKI) imatinib entered clinical trial in 1998 and became generally available over the next few years. The IRIS trial of first-line therapy recruited during 2000-01, and rapidly established that imatinib was more efficacious and less toxic than IFN. A 10-year follow-up of the imatinib recipients in this study revealed an overall survival of 83%, with approximately half remaining on imatinib throughout and 82% achieving CCyR [3]. This high cytogenetic remission rate led to the adoption of BCR-ABL1 quantitation by real-time quantitative reverse transcriptase polymerase chain reaction (PCR) for monitoring responses to TKI treatment, and prompted the introduction of deeper remission yardsticks; major molecular response (MMR or MR3; BCR-ABL1/ABL1 ratio ≤ 0.1%), MR4 (BCR-ABL1/ABL1 ratio ≤ 0.01%) and MR4.5 (BCR-ABL1/ABL1 ratio ≤ 0.0032%). There is a vogue to refer to MR4 or lower as ‘deep molecular response’.

Some patients receiving imatinib achieve not only CCyR but also molecular negativity. After an encouraging pilot study by the French CML group of stopping imatinib in 12 patients [4], this group carried out STIM, a bold study in 100 patients with undetectable disease by the molecular techniques of ~ 2006. Twelve months after stopping treatment, 41% remained molecularly negative [5], and at 5 years 38% remained recurrence free [6]. The vast majority of recurrences occurred in the first 6 months or so after stopping. This concentration of recurrences in the first few months after stopping treatment is a feature seen in virtually all subsequent studies.

## Studies of Treatment-Free Remission

Earlier [7•, 8•] and more recent [9•] studies of treatment-free remission (TFR) have been ably reviewed. Table 1 summarizes the main studies of TFR. During the course of these studies, the definition of recurrence has evolved from the emergence of BCR-ABL1 positivity as used in STIM and the almost contemporary Australian TWISTER study [10, 11], to loss of MMR. This latter definition was first used in the A-STIM study (12) which had similar entry criteria to STIM but reported a recurrence free survival (RFS) of 61% at 3 years. Loss of MMR has been widely used as the definition of recurrence in almost all the majority of more recent studies (see Table 1).

**Table 1:** A non-exhaustive list of the key studies of TKI discontinuation. CMR = complete molecular remission; DMR = deep molecular remission, as defined in the text. a: CMR was defined as undetectable BCR-ABL1 transcripts with sensitivity ≥ 40,000 amplified copies of the ABL control gene. b: All cases had an initial 12 months of half-dose treatment. c: Retrospective observational study

Study acronym	Ref	Entry requirements	No	Definition of recurrence	Recurrence-free survival after stopping	Factors predicting lower recurrence risk
STIM1^a^	[5, 6]	CMR; Undetectable for ≥ 2 yrs	100	BCR-ABL1 positivity	38% at 5 years	Lower Sokal score and longer TKI duration
TWISTER	[10, 11]	Imatinib for ≥3 yrs. Undetectable for ≥ 2 yrs	40	Loss of MMR OR 2 consecutive positives	47.1% at 2 years; 45% at 8.6 years	Prior IFN
A-STIM ^a^	[12]	CMR; Undetectable for ≥ 2 yrs	80	Loss of MMR	61% at 3 years	
STOP 2G-TKI	[13]	2G TKI ≥ 3 yrs.: MR4.5 for ≥ 2 yrs	60	Loss of MMR	54% at 4 years	Lack of TKI resistance to first line treatment
EURO-SKI	[14••]	TKI ≥ 3 yrs.; MR4 for ≥ 12 months	755	Loss of MMR	50% at 2 years	Longer treatment and DMR duration
DESTINY ^b^	[15, 16••]	TKI ≥ 3 yrs.; MR4 or MMR	174	Loss of MMR	72% (MR4)/36% (MMR) at 2 yrs	Longer treatment duration
Unnamed^c^	17	Unrestricted; ≥ MR4 on three occasions	293	Variable (retrospective)	62% at 34 months	Retrospective study, not a trial
Unnamed^c^	18	TKI ≥ 3 yrs.; MR4.5 ≥ 2 yrs	236	Loss of MMR or > 1 log rise	64% at 4 years	Retrospective study, not a trial
ISAV	19	Imatinib > 2 yrs.; CMR; Undetectable ≥ 18 months	108	Loss of MMR	48% at 3 years	Older age
KID	20	Imatinib for ≥ 3 yrs. Undetectable for ≥ 2 years	156	Loss of MMR	58.5% at 24 months	Longer duration of imatinib and presence of withdrawal syndrome
RE-STIM	21	In DMR after a first unsuccessful TFR attempt	70	Loss of MMR	35% at 3 years	Slower speed of molecular relapse after the first TKI discontinuation attempt
ENESTop	22	Imatinib then nilotinib ≥ 3 yrs.; MR4.5	126	Loss of MMR or confirmed loss of MR4	53% at 96 weeks	
DASFREE	23	Dasatinib > 2 yrs. (first-line or later); MR4.5	84	Loss of MMR	48% at 1 year	Includes patients resistant to first line TKI
DADI	24	Dasatinib as second or subsequent line; MR4	63	Loss of MR4	44% at 36 months	Lack of imatinib resistance
ENESTfreedom	25	Nilotinib ≥ 3 years (first line); MR4.5 at entry	190	Loss of MMR	48.8% at 96 weeks	
ENESTpath	26	Nilotinib (second line); MR4	620	Los of MR4	Yet to report TFR phase	
ENESTgoal	27	Nilotinib (second line); MR4.5	59	Loss of MMR	Yet to report TFR phase	(Only 17 of 59 entrants entered the TFR phase)

Most studies have focussed on patients with excellent molecular responses to their first-line TKI (usually imatinib), and report an RFS of 48–61% at 3 years of TKI discontinuation. There are considerable differences in the detailed eligibility for study entry, which complicates comparisons between studies. Most academic-led studies have used the presence of at least MR4 sustained for 1–2 years before entry, while studies led by pharmaceutical companies such as DASFREE [23] or ENESTfreedom [25] are typically more cautious, requiring MR4.5, either sustained or at least at study entry. There is no evidence that those studies restricted to patients in MR4.5 produce a better RFS than those allowing patients in only MR4. The ISAV study [19] found that TFR was more likely to be successful in older patients. The reasons for this are not clear.

Information on TFR is limited in patients resistant to a first-line TKI (almost always imatinib) who then achieve excellent responses to a second-line TKI. The ENESTop study [22] of patients on second-line nilotinib was cautious in that patients had to be in at least durable MR4.5 for entry, and maintain this on nilotinib during a subsequent 48-week observation period; it reported 53% RFS at 96 weeks after stopping. The DADI study [24] reported a RFS of 44% at 3 years after stopping second-line dasatinib, though used loss of MR4 as its definition of recurrence. The STOP-2G TKI [13] reported a RFS of 54% at 4 years using loss of MMR as its recurrence endpoint. Interestingly, both this and the DADI study reported better RFS for patients who were intolerant of first-line imatinib than for those with first-line TKI resistance. ENESTpath is in progress, comparing two different durations of second-line nilotinib before stopping treatment.

## How Might we Improve TFR Success?

In the STIM trial, a non-significant trend for a better TFR success rate was seen in patients who had previously received IFN [5], though patients in early TFR studies who had received initial IFN tended to have received their TKI for longer, and also IFN may increase the proportion of patients achieving remissions deep enough to allow consideration of TFR. In the large multinational EURO-SKI study, patients pretreated with IFN for > 1.5 years had a 6-month RFS of 86%, which was significantly higher than the 56% seen in patients who had received IFN for ≤ 1.5 years [14••]. IFN maintenance therapy may enable high rates of treatment discontinuation success [28], and the value of pegylated IFN maintenance is currently being prospectively explored in the ENDURE study (https://clinicaltrials.gov/ct2/show/NCT03117816). The German TIGER (CML V) study is currently comparing nilotinib ± IFN as first-line treatment, followed by an IFN-only maintenance phase and then a TFR attempt where appropriate. Its first results may be available in late 2019, though data on the TFR phase may take rather longer. Similarly, the Nordic and French group are examining initial bosutinib with or without pegylated IFN, followed by TFR, in the BOSUPEG trial. All these studies require stable MR4 before attempting TFR, and over the course of the next 2–3 years will establish whether concurrent and sequential IFN with TKI improves TFR success.

It is plausible that other treatment strategies might increase the TFR success rate. Recently a small study of lenolidamide as maintenance therapy after TKI withdrawal has been reported [29].

All but one of the studies summarized in Table 1 have used an abrupt discontinuation of TKI. However, it is plausible that some patients who fail to successfully stop their TKI might nevertheless remain in good remission on lower doses, which might ameliorate TKI-related adverse events and help with the burden of drug costs. Reduction of therapy appears safe [30], and mathematical modelling of clinical trial data predicts that after an initial phase of disease bulk reduction, reduced TKI doses are likely to control disease as effectively as standard doses [31]. The British De-Escalation and Stopping Treatment of Imatinib, Nilotinib or sprYcel (DESTINY) study differs from the other studies summarized in Table 1 in that patients first receive 12 months of their TKI at half the standard dose (imatinib 200 mg daily, nilotinib 200 mg twice daily or dasatinib 50 mg daily) before outright stopping. At the end of this de-escalation phase, 98% of patients in at least stable MR4 over the year prior to entry remain recurrence-free [15], and at 36 months (i.e. after a subsequent 2 years of complete cessation), the overall RFS is 72% [16••]. These excellent results have only been equalled by a small study of interferon maintenance on imatinib withdrawal [28] and recent observational retrospective studies where patient selection may have been a factor [17, 18].

It is possible that gradual TKI withdrawal might provoke hitherto quiescent leukaemic stem cells (LSC) into a more proliferative state in which they may become vulnerable to the TKI, thus lowering the LSC burden at subsequent TKI cessation. The kinetics of residual leukaemia during de-escalated TKI doses or after stopping are difficult to study, since by definition there is very little disease present, though recent data on ten patients in the DESTINY study suggest that lower marrow baseline levels of cells positive for CD93, a potential LSC marker, may predict a better RFS [32]. An alternative possibility is that gradual TKI withdrawal in some way coerces the immune system to respond to residual leukaemia. TKIs may suppress T cell proliferation and activation and dendritic, natural killer and B cell function (reviewed in [33]), but this does not appear to have significant clinical consequences. Immune responses to BCR-ABL peptide vaccination in TKI recipients are the same as in normal subjects [34].

There are recent data about how immune responses may alter after TKI withdrawal. In DESTINY patients, CD4+ regulatory T cells and CD56^dim^ and CD56^bright^ NK cell subsets fall during de-escalation, though no further lymphocyte subset changes are seen after subsequent TKI cessation; furthermore, a rise in the effector memory CD8+ subset predicts recurrence [35]. Patients in the EURO-SKI trial with higher levels of NK cells and lower CD86+ plasmacytoid dendritic cell counts at the time of treatment cessation have a higher probability of successful treatment discontinuation [36, 37], and their numbers may increase further after treatment cessation in non-relapsing patients [38]. Overall, current data suggest that the degree to which the immune system is affected by TKIs may be greater in patients with disease recurrence after TKI cessation. Although this is currently of little practical use in the clinic, further study of the immune system during TKI withdrawal will be useful. Other studies of gradual TKI withdrawal are currently being planned, and it is hoped that they will incorporate laboratory studies of the immune system.

## What PCR Level Is Required?

**Fig. 1:**
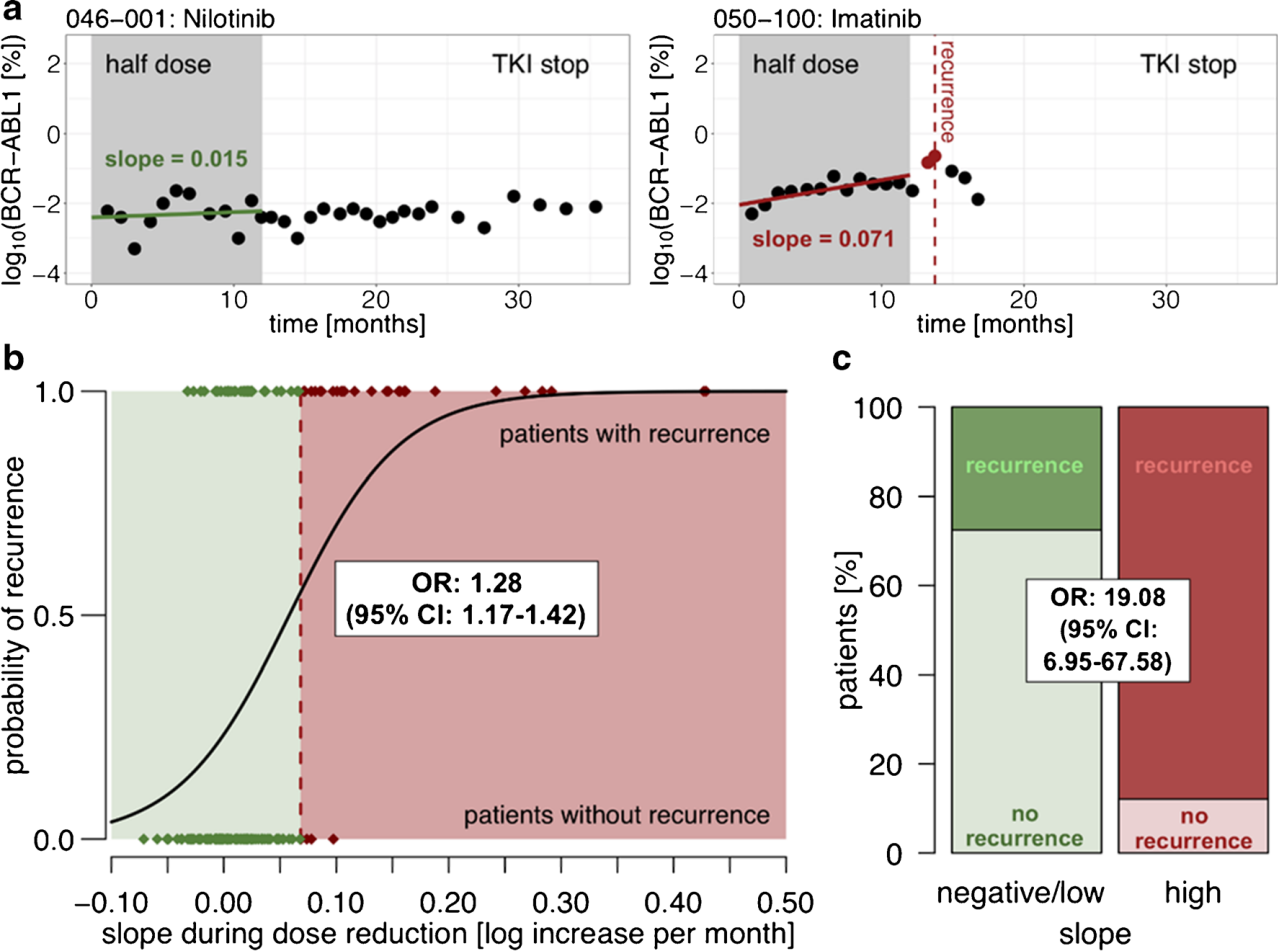
Results from reference 44 on the DESTINY trial [16••] data, showing the relationship between the slope of the BCR-ABL1/ABL1 PCR during the initial 12-month dose reduction period and the probability of recurrence. OR = odds ratio; CI = 95% confidence interval. *n* = 171 throughout. **A** PCR dynamics of two representative patients during the dose reduction period. Left: a patient with a low positive slope (0.015 log rise per month; green line) that remains in TFR after stopping TKI. Right: a patient with a high slope (0.071 log rise per month; red line); recurrence occurred shortly after stopping TKI. **B** Logistic regression curve for patient-specific estimates of the individual PCR slope during dose reduction, with corresponding OR and CI per 0.01 log-increase per month. Patients are separated into cohorts with negative/low slopes (green area) or high slopes (red area) by a cut-off of 0.068 log rise per month. **C** Proportion of patients with and without recurrence in the negative/low and high slope cohorts during dose reduction, and corresponding OR and CI

As Table 1 shows, the vast majority of studies of TFR have focussed on patients in at least stable MR4. The current National Comprehensive Cancer Network (US) guidelines recommend that TFR should only be attempted in patients who have been in at least MR4 for at least 2 years [39]. Those of the European Society for Medical Oncology recommend achievement of MR4.5 and stable in at least MR4 for at least 2 years [40], though acknowledge that less stringent criteria do not exclude successful TFR. However, absence of evidence does not constitute evidence of absence. Apart from anecdotal reports[41, 42] and a study of 12 patients [43], TFR has not been formally studied in patients achieving enduring MMR but not necessarily MR4. However, 49 patients who had received TKI for at least 3 years and were in stable MMR but not always MR4 during the previous 12 months were included in the DESTINY study cited above. Their RFS at 12 months of de-escalation was 81%, [15], and 36% at 3 years (after a subsequent 2 years of stopping) [16••]. These are inferior results to those seen in patients in at least stable MR4 (98% and 72% respectively). However, better RFS was seen in patients who had received their treatment for longer, and the RFS in the MR4 patients may be similar to MMR patients who have received their TKI for 2–3 years longer [16••]. Furthermore, the trend (slope) in the BCR-ABL1/ABL1 ratio during de-escalation may be used to predict the probability of recurrence after subsequent TKI cessation [44]. Panel A of Fig. 1 illustrates two contrasting representative patients, on the left with almost no PCR change and no recurrence and on the right with a high upward slope who did relapse. A PCR rise of greater than 0.068 log per month is associated with a high probability of recurrence on TKI stopping, and this PCR slope could be used to guide advice about PCR cessation; indeed, if TKI cessation were limited to patients with negative (falling PCR) or low positive slopes then the 2-year RFS for MMR patients exceeds 50% [40]. Overall, these data in MMR patients require confirmation in further studies, and at present it may be wise to consider TFR attempts for such patients as experimental and best confined to clinical trials.

## Other Considerations

Surprisingly, failure of a TFR attempt does not necessarily preclude success at a second attempt, though information on the best approach for such patients is currently lacking. The observational RE-STIM study, of 70 patients failing a first TFR attempt who then regained a stable remission of at least MR4, reported an RFS of 42% at 2 years after a second stop, and this may be higher in patients with longer times to recurrence in the first stopping attempt [21]. These results are not dramatically inferior to those in first TFR attempts, and it is unclear why patients who fail TFR initially may succeed with essentially the same strategy a few months later. Studies of a second TFR attempt are in progress for imatinib recipients that fail a TFR attempt, in which treatment is first changed to either nilotinib (NILO post-STIM, NAUT) or dasatinib (TRAD).

There is some evidence that RFS after stopping TKI may be better in patients with the e14a2 form of BCR-ABL1 transcript than in those with the e13a2 form [45, 46], though this finding is not universal. It is unclear whether this is real or due to a PCR artefact whereby a given level of e13a2 burden may appear higher than for e14a2, thus disproportionately lowering any relapse threshold in e13a2 patients. Almost no data are available on TFR for patients with variant BCR-ABL1 transcripts (i.e. not e13a2 or e14a2) as such patients have been excluded from most studies due to the lack of parity between PCR techniques for variant transcripts versus e13a2/e14a2 and the difficulties of standardization of variant transcript PCR techniques. As a result, several guidelines do not recommend attempting TFR in such patients.

In the past few years, new molecular platforms have become available which are more sensitive than conventional RT-PCR. These include DNA-based PCR and digital and next-generation sequencing methodology (reviewed in [47]). Using such techniques, disease may remain detectable in patients who have undetectable BCR-ABL by conventional RT-PCR [10]. This has led to the suggestion that such techniques may be useful for identifying patients at higher risk of recurrence, though few studies have investigated this possibility to date. However, many patients undergoing TFR have disease detectable by conventional PCR at TKI cessation, yet can remain off treatment for months and years without losing MMR, even if PCR levels may rise. Since such patients are clearly not leukaemia-free yet can manage without treatment, this raises questions about the clonal kinetics during TFR and at recurrence. A particular difficulty is investigating the LSC compartment during TFR, since this is by definition small, may be quiescent, and if not then it is dwarfed by its differentiated progeny.

All TFR studies to date have specifically excluded patients with prior advanced phase CML, even if in excellent TKI-induced remissions for many years, presumably on the grounds that they potentially harbour clones with the proven capability of relapsing in blast crisis. This exclusion is unlikely to change and TFR is best regarded as contraindicated for such patients.

## TKI Withdrawal Syndrome

Withdrawal symptoms on TKI cessation were first described in 2014 [48, 49] in 15 of 50 Swedish patients in the EURO-SKI study. In a retrospective analysis of STOP-TKI and EURO-SKI data, 23% of 427 patients developed musculoskeletal symptoms on TKI withdrawal [50]. These mainly affected the upper body joints, and required multiple symptomatic treatments in 30% of patients. The mechanism of this TKI withdrawal syndrome is not clear, though has been suggested to be due to withdrawal of an off-target effect (i.e. a target other than ABL1) of the TKI. All TKIs have off target effects, though the relative magnitude of these on a given target differ between the TKIs, whereas there is currently little evidence of differences in rate or clinical manifestation of the withdrawal syndrome between the TKIs. Duration of TKI treatment and a previous history of osteoarticular symptoms increased the risk of symptoms. Most subsequent studies have also reported similar symptoms in 25–40% of patients stopping TKI (reviewed in [51•]). In the KID study, its occurrence is associated with a greater TFR success rate, and a longer time to recurrence in those who do relapse [20], though this is not seen in other studies. The effect of musculoskeletal symptoms on quality of life is not known, as few studies have assessed this during TFR and these have typically used assessment instruments for general cancer that may be too insensitive for well-controlled CML patients. Symptoms may be more likely in patients with lower body weight and body mass index [52]. Although the symptoms may resemble other inflammatory arthropathies, rheumatoid factor, markers of systemic lupus erythematosus and radiological investigations for joint damage are typically negative [53].

The natural history appears to be gradual and complete resolution, though this may take many months. There are no data on optimal treatment. Symptomatic treatment with mild analgesics such as paracetamol and non-steroidal anti-inflammatory drugs may help; more severe cases may require a course of steroids. In the original Swedish series within EURO-SKI, symptoms resolved in all six patients who resumed imatinib because of recurrence [48] and this amelioration on resumption of TKI has been confirmed in other series, though patients are understandably reluctant to resume TKI solely for this reason.

## Practical Considerations

TKIs are expensive drugs, and therefore it is not surprising that both treatment de-escalation and withdrawal have been reported to save substantial sums of money [14••, 15]. This cost saving may be less dramatic since imatinib is now generic and therefore cheaper in most countries, and may diminish further as newer TKIs follow suit in the coming years. However, more frequent molecular monitoring is required for patients who stop treatment, especially in the first few months. The National Comprehensive Cancer Network (NCCN) guidelines recommend monthly testing for the first 12 months [35], and this additional monitoring partly offsets the savings on drug costs. An interesting recent theoretical report predicts that two monthly monitoring for the first 6 months and 3 monthly thereafter will still be safe enough to capture recurrence (loss of MMR) before patients lose CCyR [54]. It will be interesting to see this bold monitoring schedule applied in future TFR trial designs.

## Conclusions

It is clear that some patients with excellent responses to a few years of TKI therapy can successfully discontinue treatment. TFR appears safe; there are currently only two reports of disease progression (lymphoid blast crisis in the A-STIM study [12] and blast crisis in the KID study [55]) amongst many thousand patients attempting TFR from stable remissions of MMR or better, and almost all patients with disease recurrence after stopping TKI will regain MMR and MR4 on resuming their TKI. There is therefore an understandable current trend to regard successful TFR as the primary treatment goal, and TFR rightly features centrally in the design of the trials of most major CML groups.

However, caution is urged before adopting TFR as the sine plus ultra of CML treatment, in place of the more humble aim of at least MMR (and thus freedom from progression) without unacceptable side effects. Around 20% of otherwise eligible patients may not wish to stop treatment [16••, 56], and TKI cessation may raise levels of anxiety and depression [57]. Interestingly, a recent survey of over 1500 patients suggests that they are less concerned about TFR than haematologists, and regard other facets of the disease as of greater importance [58]. The precise definition of who might be eligible for a TFR attempt is not yet finalized, and key questions remain about the role of IFN in initial remission induction or in consolidation before attempting TFR. More work is needed on whether gradual treatment withdrawal, which clearly ameliorates symptoms, can improve TFR success, and whether TFR can be extended to patients in stable MMR but not necessarily MR4; we also do not know how best to advise patients who fail a first TFR attempt and wish to try again. Funding further investigation of TFR in a disease with survival approximating to the normal population is a challenge; nevertheless, several studies of these aspects of TFR are underway and are likely to report in the next few years.
